# Generic prediction of exocytosis rate constants by size-based surface energies of nanoparticles and cells

**DOI:** 10.1038/s41598-022-20761-z

**Published:** 2022-10-24

**Authors:** Bingqing Lu, Jiaqi Wang, Paul T. J. Scheepers, A. Jan Hendriks, Tom M. Nolte

**Affiliations:** 1 Department of Environmental Science, Institute for Biological and Environmental Sciences, Radboud University Nijmegen, 6500 GL Nijmegen, The Netherlands; 2 Department of Toxicology, Radboud University Nijmegen, 6500 GL Nijmegen, The Netherlands

**Keywords:** Nanomedicine, Nanotoxicology

## Abstract

Nanotechnology brings benefits in fields such as biomedicine but nanoparticles (NPs) may also have adverse health effects. The effects of surface-modified NPs at the cellular level have major implications for both medicine and toxicology. Semi-empirical and mechanism-based models aid to understand the cellular transport of various NPs and its implications for quantitatively biological exposure while avoiding large-scale experiments. We hypothesized relationships between NPs-cellular elimination, surface functionality and elimination pathways by cells. Surface free energy components were used to characterize the transport of NPs onto membranes and with lipid vesicles, covering both influences by size and hydrophobicity of NPs. The model was built based on properties of neutral NPs and cells, defining Van de Waals forces, electrostatic forces and Lewis acid–base (polar) interactions between NPs and vesicles as well as between vesicles and cell membranes. We yielded a generic model for estimating exocytosis rate constants of various neutral NPs by cells based on the vesicle-transported exocytosis pathways. Our results indicate that most models are well fitted (*R*^*2*^ ranging from 0.61 to 0.98) and may provide good predictions of exocytosis rate constants for NPs with differing surface functionalities (prediction errors are within 2 times for macrophages). Exocytosis rates differ between cancerous cells with metastatic potential and non-cancerous cells. Our model provides a reference for cellular elimination of NPs, and intends for medical applications and risk assessment.

## Introduction

Nanoparticles (NPs) have been extensively studied due to their artificial functionalization for biomedical development. Mediated by size and surface charge, NPs carry molecules across cell membranes to reach target organs^1^, cells or organelles. Size, surface charge and hydrophobicity have effect on the rates of transmembrane (cell membrane, mitochondrial membrane, etc.) transport efficiency^2,3^. Hydrophobicity of NPs affects bioavailability^4^ of NPs (hence, drugs) and increase the half-life of NPs circulating in the body by avoiding recognition by the reticuloendothelial system (RES)^5^. Quantifying the effects of NP surface properties on cellular elimination is of critical importance in biomedicine. NPs must be non-toxic and biocompatible to be used in biomedical applications^6^. However, the multiple pathways of NPs exposure to humans and low targeting efficiency easily lead to unexpected cytotoxicity in vitro and in vivo^7,8^. Quantifying the transport of NPs with different properties in cells can provide a reference for the assessment of toxicological risks^9^ of NPs.

Probabilistic methods, such as artificial neural network (ANN)^10^ methods, can predict dynamic equilibria cellular uptake and exocytosis rate constants of NPs disregarding mechanisms. Mechanistic models^11,12^ focus on known transport pathways of NPs transportation in organisms and can be extrapolated to predictions of other NPs but require experimental confirmation. Biological exposure and transportation of NPs relate to factors, such as charge density, surface charge of NPs^13^, and oil–water partitioning^14,15^. The octanol–water partition coefficient (*K*_*ow*_) is traditionally used to predict the accumulation/transformation of small organic compounds in environments^16^ and organisms^17^. However, NPs interact with bio-membranes in an energy-dependent manner^18^, preventing NPs to disperse in a thermodynamically stable way^19,20^. Consequently, the *K*_*ow*_ is inadequate to describe biological exposure of NPs. Mechanism-based modelling and appropriate descriptors are needed to describe NP-biological interactions.

**Figure 1:**
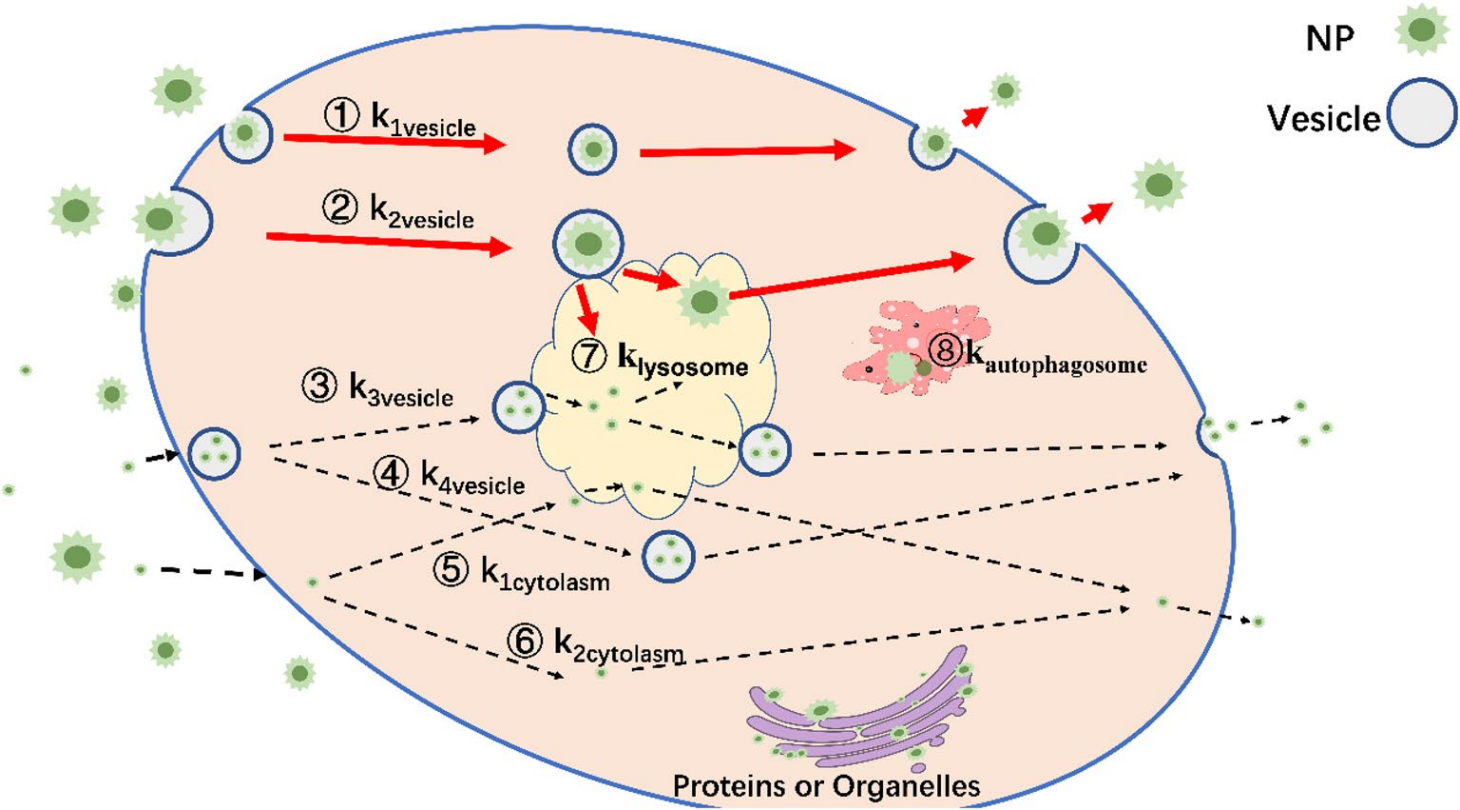
Potential elimination pathways of NPs in a cell. Pathways 1–2 (single NPs) and 3–4 (aggregated NPs) are vesicle-transported exocytosis pathways, 5 and 6 are vesicle-free exocytosis pathways, 7 and 8 are degradation pathways for lysosomes and autophagosomes, respectively. Our modelling is based on vesicle-transported exocytosis pathways for unaggregated NPs (pathways 1 and 2).

Here, we explore exocytosis rate constants of NPs based on cellular transport pathways^21^. Potential intracellular transport pathways of NPs are summarized into three categories (Fig. 1). First, NPs either encapsulated by vesicles or not, diffuse through the cytoplasm and exit cells through the membranes^22,23^, i.e., a non-endocytic pathway (e.g. red blood cells^24^). Second, NPs may be transported to lysosomes, degraded by acid (pH ~ 4–5) or not^25,26^, and finally be excreted out of cells^27^. Smaller NPs can even aggregate into a cluster and be transported by a vesicle^28^. Other pathways, include adhesion to the endoplasmic reticulum^29^, nucleus^30^ or golgi apparatus^31^, and subsequent transported by the aforementioned pathways.

NP properties affect their transport pathway and, hence, their exocytosis rates^32^. According to the conservation law, the apparent (experimental) elimination rate constant (*k*_*eli*_) is a combination (summation) of different processes. The relevance of the different pathways relates to the intrinsic rate constants (*k*) of different pathways and the corresponding NPs concentrations in the respective cellular compartments:

1 $$\begin{aligned} \left[ {{\text{NP}}} \right]_{{{\text{total}}}} \cdot k_{eli} & = \left[ {{\text{NP}}} \right]_{{{\text{vesicle}}}} \cdot k_{vesicle} + \, \left[ {{\text{NP}}} \right]_{{{\text{cytoplasm}}}} \cdot k_{cytoplasm} + \, \left[ {{\text{NP}}} \right]_{{{\text{lysosome}}}} \cdot k_{lysosome} \\ & \;\;\; + \left[ {{\text{NP}}} \right]_{{{\text{autophagosome}}}} \cdot k_{autophagosome} + \ldots \\ \end{aligned}$$

Or

2 $$\begin{aligned} k_{{eli}} ~~ & = \left( {\left[ {{\text{NP}}} \right]_{{{\text{vesicle}}}} /\left[ {{\text{NP}}} \right]_{{{\text{total}}}} } \right) \cdot k_{{vesicle}} \left( {{\text{pathways}}\;{\mathbf{1}} - {\mathbf{4}}} \right) \\ & \;\;\; + \left( {\left[ {{\text{NP}}} \right]_{{{\text{cytoplasm}}}} /\left[ {{\text{NP}}} \right]_{{{\text{total}}}} } \right) \cdot k_{{cytoplasm}} \left( {{\text{pathways}}\;{\mathbf{5}}\;{\text{and}}\;{\mathbf{6}}} \right) \\ & \;\;\; + \left( {\left[ {{\text{NP}}} \right]_{{{\text{lysosome}}}} /\left[ {{\text{NP}}} \right]_{{{\text{total}}}} } \right) \cdot k_{{lysosome}} \left( {{\text{pathway}}\;{\mathbf{7}}} \right) \\ & \;\;\; + \left( {\left[ {{\text{NP}}} \right]_{{{\text{autophagosome}}}} /\left[ {{\text{NP}}} \right]_{{{\text{total}}}} } \right) \cdot k_{{autophagosome}} ({\text{pathway}}\;{\mathbf{8}}) + \cdots \\ \end{aligned}$$

where [NP]_vesicle_ and [NP]_total_ are the concentration of vesicle-transported NPs and total NPs inside of one cell, respectively. [NP]_vesicle_/[NP]_total_ is the fraction of NPs encapsulated by vesicles. Degradation (including *k*_lysosome_^29^ and *k*_autophagosome_^33^) are part of elimination pathways in cells (e.g., dissolution of NPs), depending on enzyme properties^34^, NP properties, such as coating stability^35^, surface hydrophobicity^36^ and charge^37^, and inner core materials^29^. It is difficult to qualify the degradation of NPs directly (pathways 7 and 8). Lysosomes ‘modification’^38^ or aggregation^19^ of NPs can render the (new) surface charge and structure of NPs uncertain, especially for charged NPs.

### Hypothesis/scope

Figure 1 depicts the possible cellular elimination and transport pathways of vesicle-transported NPs and vesicle-free NPs inside or outside cells. By considering inert NPs we minimized the effects of acidification within lysosomes on surface modifications, and thereby built and parameterized a generic exocytosis model based on exocytosis pathways for single NPs (Fig. 1 pathways 1 and 2):

3 $$k_{exo} = \left( {\left[ {{\text{NP}}} \right]_{{{\text{vesicle}}}} /\left[ {{\text{NP}}} \right]_{{{\text{total}}}} } \right) \cdot k_{vesicle}$$

Figure 1 depicts six exocytosis pathways (1–6). It is difficult to define the proportion of vesicle-transported NPs ([NP]_vesicle_/[NP]_total_) for our generic modelling due to rather limited data. Previous studies tried to predict transportation of NPs based on the deformation^39^ or combination^40^ energies, which relates to NPs being encapsulated by vesicles to cross cell membranes. Correspondingly, deformation energies during cell membranes and vesicles recombination drive exocytosis of NPs^41^, but predicting probability/frequency of vesicle-transported NPs^42^ remains cumbersome.

In this paper, we developed a generic model to predict the exocytosis rate constants (*k*_*exo*_) by calculating the interaction between vesicle-transported NPs and cell membranes based on properties of NPs and traits of cells. We considered the fraction or frequency of NPs encapsulated by vesicles to predict exocytosis of NPs by cells. We focused on Van de Waals, electrostatic and Lewis acid–base (polar) forces during the interaction of vesicle-transported NPs with cell membranes. We applied our modeling framework to inert neutral particles to avoid the influence caused by acidic environments in lysosomes. The experimental data of 64 types of NPs and six types of cell lines were used as training and test data to predict the exocytosis rate constants.

## Results

### Interaction energies between vesicle-transported NPs and cell membranes

Figure S1 depicts the total energies changes over the distance among NPs, vesicles and cells. Table 1 shows all interaction energies between NPs and vesicles (Δ*G*_np/v_) and energy barrier heights between vesicles and membranes (Δ*G*^‡^_v/m_). All the interaction energies and barrier energies are positive values. The Δ*G*_np/v_ between different sized vesicle-transported NPs (size from 14 to 100 nm) and vesicles range from 350 to 3400 kJ/mol. The Δ*G*^‡^_v/m_ between vesicles (size from 14 to 100 nm) and cell membranes range from 600 to 4600 kJ/mol, which is always higher than the Δ*G*_np/v_ between NPs and vesicles, except for citrate- and PEG-coated NPs.

**Table 1:** The interaction energies between NPs and vesicles and energy barriers heights between vesicles and cell membranes for 64 organically-coated gold NPs.

No	NPs	Cells	ΔG_np/v_ kJ/mol	ΔG^‡^_v/m_ kJ/mol	No	NPs	Cells	ΔG_np/v_ kJ/mol	ΔG^‡^_v/m_ kJ/mol
1	C1(methoxy)	RAW 264.7	779	1171	33	C12B(2-butyloctyl)	Hela	453	1270
2	C6S(hexyl)	RAW 264.7	571	1185	34	C12C(cyclododecyl)	Hela	436	1194
3	C6B-2(3,3-dimethylbutyl)	RAW 264.7	579	1199	35	C12A-1(5-naphthybutyl)	Hela	505	1250
4	C6C(cyclohexyl)	RAW 264.7	577	1165	36	C12A-2(7-phenylheptyl)	Hela	458	1199
5	C6E-1(5-hexenyl)	RAW 264.7	570	1165	37	Citrate	U937	675	667
6	C6E-2(2-hexenyl)	RAW 264.7	607	1237	38	Citrate	U937	1259	1243
7	C6A(phenyl)	RAW 264.7	682	1302	39	Citrate	U937	2132	2105
8	C12S(dodecyl)	RAW 264.7	455	1274	40	Cysteamine	U937	346	624
9	C12B(2-butyloctyl)	RAW 264.7	453	1270	41	Cysteamine	U937	744	1343
10	C12C(cyclododecyl)	RAW 264.7	436	1194	42	Cysteamine	U937	1225	2212
11	C12A-1(5-naphthybutyl)	RAW 264.7	505	1250	43	L-cysteine	U937	591	753
12	C12A-2(7-phenylheptyl)	RAW 264.7	458	1199	44	L-cysteine	U937	990	1260
13	C1(methoxy)	C166	779	1171	45	L-cysteine	U937	1776	2260
14	C6S(hexyl)	C166	571	1185	46	PEG	U937	1266	1177
15	C6B-2(3,3-dimethylbutyl)	C166	579	1199	47	PEG	U937	1789	1663
16	C6C(cyclohexyl)	C166	577	1165	48	PEG	U937	2746	2552
17	C6E-1(5-hexenyl)	C166	570	1165	49	Transferrin	STO	477	648
18	C6E-2(2-hexenyl)	C166	607	1237	50	Transferrin	STO	1022	1388
19	C6A(phenyl)	C166	682	1302	51	Transferrin	STO	1703	2314
20	C12S(dodecyl)	C166	455	1274	52	Transferrin	STO	2520	3425
21	C12B(2-butyloctyl)	C166	453	1270	53	Transferrin	STO	3406	4628
22	C12C(cyclododecyl)	C166	436	1194	54	Transferrin	SNB19	477	648
23	C12A-1(5-naphthybutyl)	C166	505	1250	55	Transferrin	SNB19	1022	1388
24	C12A-2(7-phenylheptyl)	C166	458	1199	56	Transferrin	SNB19	1703	2314
25	C1(methoxy)	Hela	779	1171	57	Transferrin	SNB19	2520	3425
26	C6S(hexyl)	Hela	571	1185	58	Transferrin	SNB19	3406	4628
27	C6B-2(3,3-dimethylbutyl)	Hela	579	1199	59	Transferrin	Hela	477	648
28	C6C(cyclohexyl)	Hela	577	1165	60	Transferrin	Hela	1022	1388
29	C6E-1(5-hexenyl)	Hela	570	1165	61	Transferrin	Hela	1703	2314
30	C6E-2(2-hexenyl)	Hela	607	1237	62	Transferrin	Hela	2520	3425
31	C6A(phenyl)	Hela	682	1302	63	Transferrin	Hela	3406	4628
32	C12S(dodecyl)	Hela	455	1274	64	D-penicillamine	Hela	248	370

### The prediction of exocytosis rate constant k_exo_

**Figure 2:**
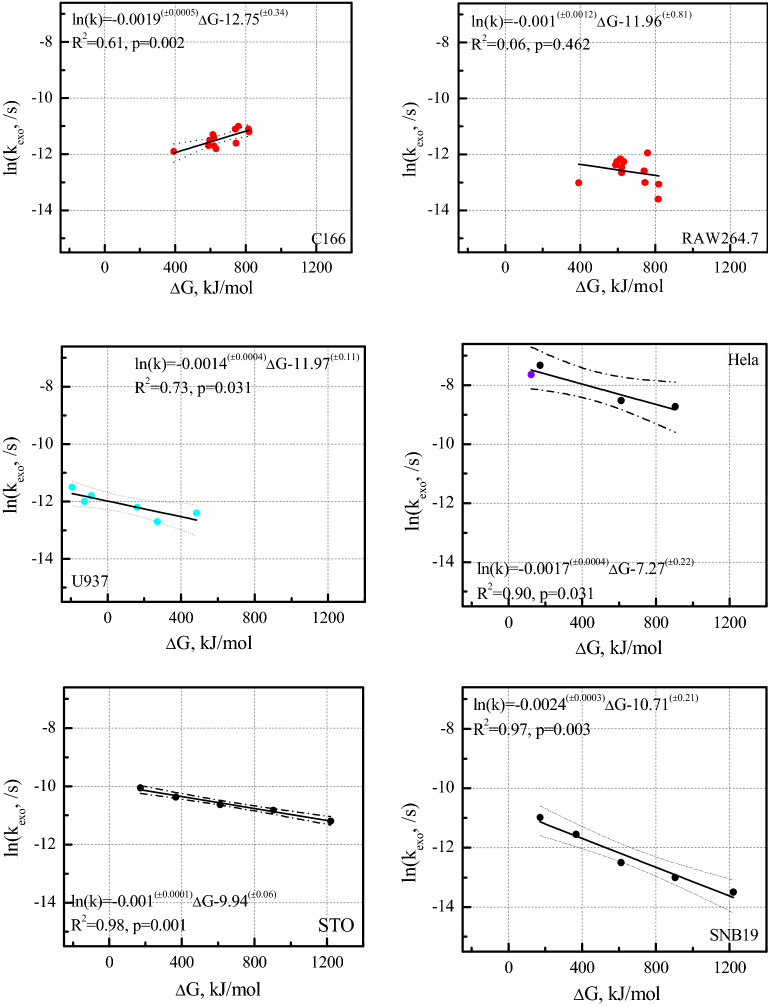
The exocytosis rate constant ln(*k*_*exo*_) (/s) vs Δ*G(d)* (Eq. 6, kJ/mol) for six cell lines (C166 and RAW264.7 (12 neutral NPs with similar sizes and different coatings, are denoted by ‘red circle’^43^. All *k*_*exo*_ were obtained based solely on initial and final intracellular NP concentrations, Table S1); U937 (three zwitterionic and three PEG-coated NPs with different sizes, are denoted by ‘blue circle’^44^. All *k*_*exo*_ were obtained based on exocytosis curves, Fig. S2); Hela (three transferrin-coated NPs with different sizes, are denoted by ‘black circle’^28^ and one D-penicillamine coated NP, is denoted by ‘purple circle’^45^. All four *k*_*exo*_ were obtained based on exocytosis curves, Fig. S2); STO and SNB19 cells (five transferrin-coated NPs with different sizes, are denoted by ‘black circle’^28^. All 10 *k*_*exo*_ were obtained based solely on initial and final intracellular NP concentrations, Table S1)). The dotted lines denote 95% conference intervals. Modelling results include linear functions, *R*^2^ and *p*-values.

The energy changes (Δ*G(d)*, Eq. 6) and experimental rate constant ln(*k*_*exo*_) for NPs exocytosis by different cells are in Fig. 2. There are explicit positive correlations between Δ*G(d)* (Eq. 6) and experimental exocytosis rate constant ln(*k*_*exo*_) for non-cancerous cells (C166 cell, Fig. 2; *R*^2^ = 0.61; and *p* = 0.002), and explicit negative correlations between Δ*G(d)* and ln(*k*_*exo*_) for non-cancerous cells (U937 and STO cells). Interestingly, cancerous cells (Hela and SNB19 cells) gave negative correlations (with *R*^2^ and *p* 0.90;0.031 and 0.97;0.003, respectively), but their slopes (*β*, Eq. 4) are lower than non-cancerous cells (U937 and STO cells), i.e. *β*_STO_ > *β*_U937_ > *β*_Hela_ > *β*_SNB19_, corresponding to − 0.0010(± 0.0001) > − 0.0014(± 0.0004) > -0.0017(± 0.0004) > − 0.0024 (± 0.0003). The correlation between Δ*G(d)* (Eq. 4) and ln(*k*_*exo*_) of RAW264.7 cells (is not significant (*p* = 0.46), due to one potential outlier. Figure 2 indicates that Hela cells have somewhat higher experimental exocytosis rate constants than any other five cells within the Δ*G* range of – 250 to 1500 kJ/mol. The frequency factors (*A*) of the six cells are also different, the lowest is the C166 cells (e^− 12.8 (± 0.3)^) and the highest is the Hela cells (e^− 7.3 (±0.2)^).

### Validation of modelling

**Figure 3:**
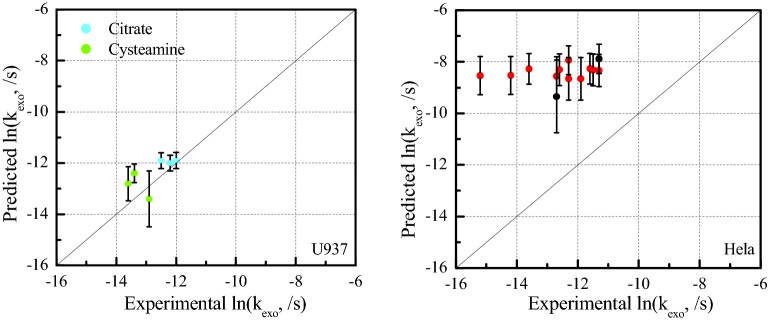
Plot of ln(*k*_*exo*_, /s) predicted by U937 and Hela models (Fig. 2) vs. experimental ln(*k*_*exo*_, /s): six ln(*k*_*exo*_, /s) based on experimental exocytosis curves of charged NPs were denoted as ‘green circle or blue circle’ (three are cationic and three are anionic^44^) for U937 cells and 14 ln(*k*_*exo*_, /s) of neutral NPs^28,43^ based solely on initial and final intracellular NP concentrations for Hela cells were denoted as ‘red circle’. The 95% confidence intervals for the predicted results are also represented by different bars.

*k*_*exo*_ for six charged NPs and 14 neutral NPs were used to test our models for U937 cells and Hela cells. Figure 3 presents that 95% confidence intervals of predicted *k*_exo_ for one cationic and two anionic NPs overlap with experimental *k*_*exo*_ while *k*_*exo*_ of two cationic and one anionic NPs were overestimated. The 95% observation-based confidence intervals of cationic NPs are wider than anionic NPs, and cationic NP at 48 nm has the largest confidence intervals. Our model (parametrized using exocytosis curves) overestimated 14 values for *k*_*exo*_ of neutral NPs that were based solely on initial and final intracellular NP concentrations in Hela cells. All 13 data have similar confidence intervals. Predicted *k*_*exo*_ values for neutral NP with size of 100 nm showed the largest confidence intervals.

## Discussion

### Modeling assessment

The calculated interaction energies (Δ*G*_np/v_) and energy barrier heights (Δ*G*^‡^_v/m_) in Table 1 are all positive, meaning that all interaction forces among NPs, vesicles and cell membranes would be repulsive. Earlier research reported that NPs with hydrodynamic sizes of 6–7 nm^46^ and AuNPs^47^ of 2 nm permeate and absorb through/by membranes with energy barrier heights of 50–500 kJ/mol. Larger aggregated NPs caused higher energy barrier heights (600–1700 kJ/mol)^47^, which are similar to the interaction energies obtained from our model. All Δ*G*^‡^_v/m_ are larger than Δ*G*_np/v_ except citrate/PEG-coated NPs^44^. This means that the large repulsive forces between the hydrophobic NPs (citrate/PEG-coated NPs) and the vesicles make it difficult for the NPs to bind to the vesicles, resulted a lower frequency of NPs being encapsulated by vesicles.

Our model is able to predict the effect of coatings of NPs with similar sizes on *k*_*exo*_ by C166 cells and effect of NP sizes with the same coating on *k*_*exo*_ by STO and SNB 19 cells. In other words, the model describes both the role of coating and size on exocytosis. Experimental exocytosis data or five cell types fit the prediction, except for RAW 264.7 cells. Five regressions had acceptable results with *p* < 0.05 and *R*^2^ ranging from 0.61 to 0.98 for C166, U937, Hela, SNB 19 and STO cells. Figure 2 showed half of the data outside the 95% confidence intervals for C166 cells, which means the model based on first-order kinetics may need further consideration. Near-perfect regressions for SNB 19 and STO cells were obtained as the experimental data have a linear relationship with surface area as reported^28^, which were also shown by our models. The result for macrophage (RAW 264.7) cells had low statistical significance (*R*^2^ = 0.06, *p* = 0.462), due to one potential outlier (Fig. 2). Possibly, our generic description of cells (*γ*, *ψ*, etc.) do not apply to abnormally proliferating blood macrophages. Errors in individual data (the single datapoint for RAW cells) may also exist due to experimental quirks: for example, lysosome-related demethylation may be particularly important in macrophages^48^.

### Frequency factors A

Most regression equations for various cells showed different slopes *β* and intercepts *A* after application of generic cellular descriptors (*γ*^LW^_cell_, *γ*^AB^_cell_ and ${\psi }_{0,cell}$). According to our hypothesis, *A* will apply to a single cell type. Since we cannot obtain *A* values based on current data, generic *A* values for each type of cells were obtained from those regressions. The six *A* values range from 2.9 × 10^–6^ to 7.0 × 10^–4^/s. Hela cells entailed the largest frequency factor (*A* = 7.0 × 10^–4^ (e^− 7.3 (± 0.2)^)) and higher experimental *k*_*exo*_ than other five cells (Fig. 2). According to our assumption, a higher *A* means a higher frequency of combination between NPs and vesicles. Reported experiments observed various NPs transport based on vesicles^28,45,49^, which confirms the model's prediction that NPs have a high frequency of exiting Hela cells via vesicles. We collected the initial uptake numbers of NPs per cell surface area (SI Table S2), and found U937 cells contained the highest number of NPs (14–610 per μm^2^) at the start of exocytosis experiment, although we got a low frequency of combination between NPs and vesicles (*A* = 6.3·10^–6^/s) for U937 cells based on our regression. As Nuri and Park reported^44^, PEG-coated gold NPs can migrate in the cytoplasm in the form of individual particles without vesicles, confirming that PEG-coated NPs avoid vesicles when entering cells. The large repulsive interaction energy between strong hydrophilic NPs and vesicles also verified this. These combined notions show that our model can describe the exocytotic behavior of differently coated NPs and the possibility of vesicle transport. In addition, protein corona may influence the distribution of NPs in organisms^50^, influencing *A*. The hydrophobicity of NP coating may correlate with antibody binding^51^. Given that hydrophobicity has already been involved in this model, our generic model ignored effects of proteins corona on NPs during exocytosis.

### Free energy slope β

Our fitted value for *β* involving C166 cells is positive, different from negative *β* involving the other four cells (U937, Hela, STO and SNB19 cells). *β* values for two cancerous (Hela and SNB 19) cells are negative: this may be related to the hydrophilic character of cancerous cells with metastatic potential facilitating these cells’ translocation to different tissues^52^. Similar to cancerous cells, STO and U937 cells have high metabolic activity and migration frequency for immune response to tissue damage^53^, which differs from somewhat ‘tranquil’ or ‘sluggish’ endothelial cells. The energy barriers (Δ*G*^‡^_v/m_) between hydrophilic vesicles and membranes for cancerous cells (Hela and SNB 19 cells) and metastatic cells (STO and U937) may therefore be underestimated (see SI methods), because we used generic cellular surface free energies (γ, ψ) for all cells to build our models. We note also that cancerous cells usually express enhanced intracellular signaling due to e.g., increased vesicle production and excretion^54^. This may result in unexpected exocytosis patterns as compared to non-cancerous cells, i.e., affect slopes *β*. Diversity of proteins in biological media^55^ will influence the surface properties both of NPs and their interaction with cell membranes. For example, cancerous cells experience contact inhibition of locomotion (CIL)^56^ behavior due to lack of cadherin, which increases repulsive forces as compared to non-cancerous cells^57^. This in turn increases energetic barriers between cancerous vesicles and cell membranes, affecting *β*. Moreover, the surface roughness or curvature of cells would influence cells surface energy^58^: macrophages have irregular shapes but that changes after cellular uptake of NPs, resulting in different curvature and surface free energy^59^. The various *β* values of specific cells embed these factors.

### Validation of modelling

Figure 3 shows that predicted *k*_*exo*_ of six charged NPs for U937 cells are close to experimental *k*_*exo*_ based on exocytosis curves. The margin of prediction error is within a factor of two (the range from 8 to 184%), demonstrating that our models based on neutral data still have the potential to predict exocytosis rate constants of charged NPs. Although *k*_*exo*_ of two cationic NPs were overestimated. Our model does not consider the aggregation of cationic NPs. However, possible aggregation of cysteamine-coated NPs was observed^44^, causing a larger effective permeant size and underestimation of Δ*G(d)*. This would imply a lower predicted *k*_*exo*_ than based on the regression between Δ*G(d)* and ln(*k*_*exo*_) (Fig. 2, U937 cells). The interaction between cationic NPs and lysosomes could change the surface energy of weakly basic NP coatings in acidic environment as well. The prediction for the smallest citrate-coated NP has the highest margin of error (33%) in comparison to the other two anionic NPs. Charge-mediated exocytosis was demonstrated in previous QSAR modelling^13^. Although we obtained good prediction for some charged NPs, data from another dataset or experiments are needed to update this generic model and incorporate the complex effects of charged NPs on exocytosis in the future.

For Hela cells, all 14 values for *k*_*exo*_ based solely on initial and final intracellular NP concentrations were overestimated (Fig. 3). The prediction errors are within 1–2 orders of magnitude. *k*_*exo*_ predicted based on kinetic exocytosis curves (Fig. S2) are far larger than ‘experimental’ *k*_*exo*_ obtained solely using initial and final intracellular NP concentrations. Both for U937 cells and Hela cells, the test NPs with largest Δ*G* showed the largest 95% confidence intervals, because its *ΔG* has exceeded *ΔG* of training (modelling) data. More data and research based on exocytosis curves for different cells are needed for updating and testing our model.

### The relationship between surface energies and partition coefficients of coating compounds (K_ow_)

**Figure 4:**
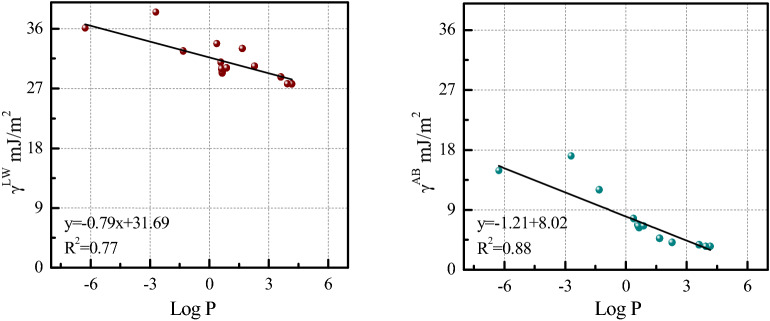
The Van der Waals free energies (γ^LW^, mJ/m^2^) and acid–base free energies (γ^AB^, mJ/m^2^) of coating compounds versus log (*K*_*ow*_ or *P*) of coating compounds.

In this paper, we used size and surface energies (mJ/m^2^) of coated NPs and cell (lipid) membranes, to calculate their interaction energies. Distinct from partition coefficients (*K*_*ow*_), surface energies^60^ describe adhesion (strength) between cells and NP materials based on surface area, which can avoid the assumed shortcomings of *K*_*ow*_ like those for characterizing NPs aggregation^19^. Yet, Fig. 4 shows strong correlations between log (*K*_*ow*_* or P*) of coating compounds and surface energy components (Van der Waals free energies (*γ*^LW^) and polar acid–base free energies (*γ*^AB^)) of coating compounds for different coatings from four datasets, confirming mechanistic similarity^61^ between *γ* values and *K*_*ow*_ both for representing hydrophobicity. Figure 4 also indicates that polar surface free energies (*γ*^AB^) have a higher correlation (*R*^*2*^ = 0.88) with log*K*_*ow*_. Our results verified that surface energy components (mJ/m^2^) characterize surface hydrophobicity of particles with specific size. Hence, our study recommended surface energy components as available descriptors to estimate NPs exocytosis and bio-transport.

### Potential and application of model

The generic model, applying descriptors of coated NPs (size, surface energy components and surface potential) and cells (surface energy components and surface potential), predict exocytosis rate constants of various NPs by calculating interactions between NPs and membranes. The model may find use in biomedicine via cellular or mitochondrial targeting^62^ by NPs or evaluating their antioxidant and anti-genotoxic capacity^63^. Our model suggests that transport experiments of NPs in the future can provide accurate surface chemical information to provide sufficient data for prediction of NPs accumulation in cells or organs, e.g., PBPK modeling^17^.

Strongly hydrophilic (e.g., PEG-coated) NPs are unlikely to bind to membranes and access vesicle transport machinery inside the cell. Nonetheless, our model still predicts the exocytosis of these PEG-coated NPs well (prediction errors within a factor two) and yields lower probabilities of NP-vesicle binding, illustrating the broadness of the model in describing hydrophobicity of different particles. Models were successfully applied to gold NPs and quantum dots with coatings, and can predict the transmembrane transport efficiency of biochemically inert NPs used for delivery of (hydrophobic) drugs, radiopharmaceuticals^5^ and nanoantioxidants to optimize biomedical applications.

Exocytosis rate constants of transferrin-coated NPs for STO cells and SNB19 cells correlate well with the NPs size as reported^28^, but this may not be true for other cell types and other NPs coatings. Our results confirm that our model can describe both effects of coatings and sizes on exocytosis, predicting exocytosis rate constants of NPs for both non-cancerous cells and cancerous cells, quantifying NPs transport for medical and toxicological applications.

Our models are built using data for neutral NPs with coating surface inert to acidic lysosomes^64^, minimizing surface modifications by lysosomes. Figure 3 shows acceptable prediction for charged NPs and U937 cells, which supports potential applicability to charged NPs. However, changes of charged, ionizable and polarizable NPs in surface free energies and surface potential after modifications within lysosomes need to be considered. Elucidating the mechanisms and behaviors of charged NPs need more experimental data. Our generic model currently does not consider aggregation and agglomeration of NPs in cells and vesicles, and a single NP is assumed to be encapsulated by a vesicle. Aggregation and agglomeration of NPs may change the properties of the whole cluster, requiring future research.

## Methods

### Model outline

**Figure 5:**
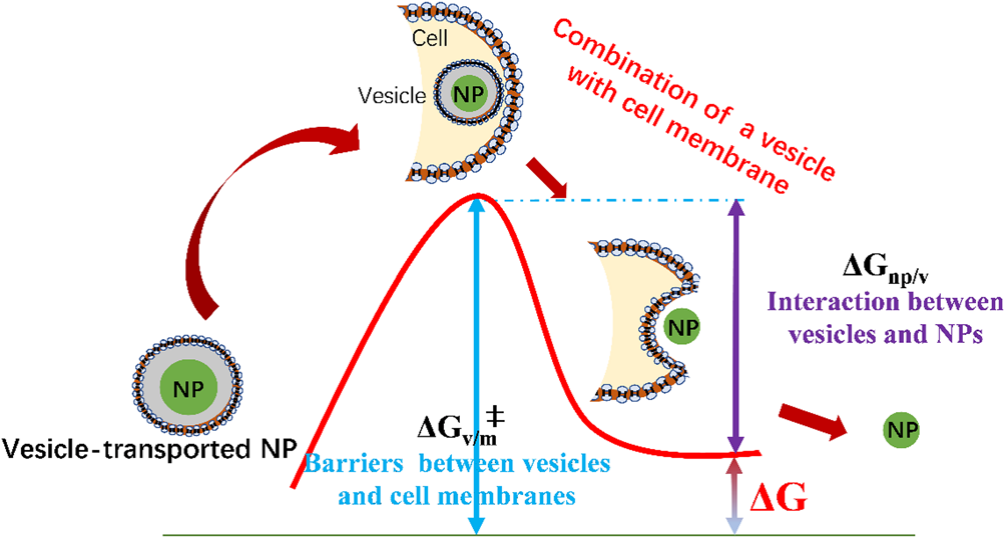
Energy profiles before and after combination of vesicles with cell membranes. Before the combination of vesicle-transported NPs with cell membranes, the energy barrier (*ΔG*_*v/m*_^‡^) between vesicles and cell membranes prevent the two from combining. At the moment of combination, the interaction (repulsive) energy (*ΔG*_*np/v*_) between the NPs and the vesicles promote the NPs to desorb from the membrane to detach. *ΔG* is the energy change at the combination moment (*ΔG*_*v/m*_^‡^ minus *ΔG*_*np/v*_).

As obtain ligand-receptor binding and deformation energy directly is difficult, we built a model based on non-covalent energy changes Δ*G* as predictors during exocytosis. We attempted to build relationships between Δ*G* values and exocytosis rate constants (*k*_*exo*_, /s) via Arrhenius equation:

4 $${\text{ln}}\left( {k_{exo} } \right) = \beta \cdot\Delta G\left( d \right) + {\text{ln}}A$$

with *β* as a regression coefficient. We fitted NPs exocytosis rate constants to energies based on vesicle-transported exocytosis for obtaining ‘generic’ values *β* (Eq. 4) applicable to different types of NPs and cells. *A* is frequency factor with the same unit of *k*_*exo*_, and energy changes are Δ*G(d)*

5 $$\Delta G\left( d \right) \, = \Delta G\left( {d_{vesicle - cell \, \;membrane} } \right) - \Delta G(d_{NP - vesicle} )$$

as a function of distances *d* between NPs, vesicles and cell membranes. Derivations below (Eq. 6) show that the two righthand terms are equal to barrier/‘activation’ (positive) energies *ΔG*_*vesicle-cell membrane*_^‡^ (*ΔG*_*v-m*_^‡^)and binding or repulsive energies *ΔG*_*np-vesicle*_ (*ΔG*_*np-v*_). Figure 5 shows the energy changes before and after exocytosis based on vesicle-transport pathways. Vesicle-transported NPs will overcome barriers to combine vesicles with cell membranes. As such, NPs are successfully excreted by the ‘release’ of the (repulsive) interaction energy between NPs and vesicles. Thereby, Δ*G(d)* are the energy changes during exocytosis process and obtained via:

6 $$\Delta G\left( d \right) \, = \, \Delta G_{vesicle - cell \, membrane^{\ddag }} - \Delta G_{np - vesicle}$$

We calculated Δ*G* between NPs and vesicles as well as between vesicles and cell membranes as a function of *d* (trajectory of exocytosis) from interaction forces involving Van de Waals (Δ*G*_*LW*_), electrostatic (Δ*G*_*e*_) and Lewis acid–base (polar) forces (Δ*G*_*AB*_) (see SI Methods)^65,66^, according to

7 $$\Delta G\left( {d_{NP - vesicle} } \right) = \Delta G_{LW} (d_{NP - vesicle} ) + \Delta G_{e} (d_{NP - vesicle} ) + \Delta G_{AB} (d_{NP - vesicle} )$$

8 $$\Delta G\left( {d_{vesicle - cell} } \right) = \Delta G_{LW} (d_{vesicle - cell} ) + \Delta G_{e} (d_{vesicle - cell} ) + \Delta G_{AB} (d_{vesicle - cell} )$$

We took the maximum interaction energy between NPs and vesicles as their interaction energy (see energy profiles in Fig. S1). All *k*_*exo*_ data were obtained from exocytosis experiments for different NPs and cells. The properties of NPs (e.g., size, surface free energies) and cells/vesicles (surface potential and surface free energies) determine the computed Δ*G(d)*. We assumed that vesicles share the same characteristics of cell membranes because vesicles combine with cell membranes during exocytosis. Additionally, we assumed that properties of neutral NPs do not change (i.e., are inert) when vesicle-transported NPs enter the lysosome’s acidic environments. Linear regressions between Δ*G(d)* and ln*k*_*exo*_ were assessed based on *R*^2^ and *p* values. A *p*-value less than 0.05 was considered to be statistically significant. The 95% confidence intervals of linear relationships (Eq. 4) were obtained via Eqs. S4–S6.

### Arrhenius equations

We stipulate that exocytosis rate constants can be described via interaction energy changes Δ*G* and the Arrhenius equation according to

9 $$k_{vesicle} =_{ } A_{vesicle} \cdot {\text{exp}}\left( { - \Delta G(d)/{\text{k}}_{{\text{B}}} {\text{T}}} \right)$$

in which *A*_*vesicle*_ is a frequency factor for vesicle-transported NPs, *A*_*vesicle*_ has the same unit as the rate constant *k* (/s). k_B_T is the product of Boltzmann's constant k_B_ and temperature T (310 K). Δ*G(d)* (Fig. 2) is the energy change before and after vesicle-transported exocytosis.

Hence, exocytosis rate constant can be derived via combining Eqs. (3) and (9):

10 $$k_{{exo}} ~ = {\text{ }}\left( {\left[ {{\text{NP}}} \right]_{{{\text{vesicle}}}} /\left[ {{\text{NP}}} \right]_{{{\text{total}}}} } \right) \cdot A_{{vesicle}} \cdot {\text{exp}}\left( { - \left( {\Delta G_{{v/m}} ^{\ddag } - \Delta G_{{np/v}} } \right)/{\text{ k}}_{{\text{B}}} {\text{T}}} \right)$$

where *A*_*vesicle*_ is the frequency factor for the vesicle-transported exocytosis pathway.

### Calculation and treatment of frequency factors A

NPs may be trafficked by cellular machinery with or without vesicles. Presumably, exocytosis rate constants are proportional to the frequency of those NPs binding to vesicles^38^. We assumed that a single NP binds to/within a vesicle^42,67^, and ignored individual reports of (intracellular) aggregation^28^ (pathway 3 and 4, Fig. 1).

Values of *A* present total frequency factors of NPs encapsulated by vesicles or vesicle-free NPs trafficked in cytoplasm. We describe frequencies as

11 $$A = A_{vesicle} \cdot \left( {\left[ {{\text{NP}}} \right]_{{{\text{vesicle}}}} /\left[ {{\text{NP}}} \right]_{{{\text{total}}}} } \right)$$

A large *A* describes a high intracellular trafficking efficiency (e.g., including production) of vesicles in the cell. It is hard to find good estimates for *A* or *A*_*vesicle*_ for specific NPs (coatings) from exocytosis experiments that ignores differences in transport pathways. We overcome this by fitting NPs exocytosis rate constants to energies during exocytosis for obtaining ‘generic’ values *A* (Eq. 4) applicable to different types of NPs and cells.

### Calculation of concentrations via Boltzmann

NPs interact with cell membranes to be encapsulated and transported by vesicles and released into the cytoplasm. Energy changes e.g. $\left( {k\, \sim \,{\text{e}}^{{ - \Delta {\text{G}}/{\text{k}}_{{\text{B}}} {\text{T}}}} } \right)$^68^ can be used to predict concentrations and transport rates of NPs through membranes, where ΔG denote the free energies along the translocation pathway. We thus calculated [NP]_vesicle_/[NP]_total_ as the relative concentrations of NPs across the cellular compartments (vesicles/cytoplasm) according to Δ*G* values:

12 $$\left[ {{\text{NP}}} \right]_{{{\text{vesicle}}}} /\left[ {{\text{NP}}} \right]_{{{\text{total}}}} = {\text{ e}}^{{ - \Delta G{\text{np}} - {\text{vesicle}}/{\text{k}}_{{\text{B}}} {\text{T}}}}$$

In which *ΔG*_np-vesicle_ is the interaction energy between NP and vesicle. ΔG_np-vesicle_ involved in the binding of NPs and vesicles, include ligand-receptor binding^69^, elastic or deformation energy^70^, and ‘generic’ surface-based DLVO (Van de Waals, electrostatic and Lewis acid–base (polar) forces). It is cumbersome to obtain ligand-receptor binding and deformation energy directly, and experimental values for *β* of different cells are not available. We overcome this by fitting NPs exocytosis rate constants to Δ*G(d)* to obtain ‘generic’ values for *β* (Eq. 4) applicable to different types of NPs and cells.

### Dataset

We applied 64 NPs with different surface modification from four open literature^28,43–45^ for experimental exocytosis to parametrize and build our models as training data and test data. 36 NPs from the first dataset (Au@PEG-X NPs) were selected with 20 nm AuNPs and functionalized with hydrocarbyl groups (X contains one, six, nine or 12 carbons) linked by polyethylene glycol (PEG), and with hydrodynamic diameter (HD, in water) of 25.17–28.13 nm^43^. The exocytosis experiments were based on 6 h intracellular uptake.

The second dataset includes four AuNPs types coated with zwitterionic cysteine, polyethylene glycol (PEG), anionic citrate and cationic cysteamine. The HD of each type NP range from 14 to 56 nm. The aggregation of NPs may happen on cationic NPs^44^. The exocytosis experiments were based on cellular uptake after 6 h at 37 °C.

The third dataset entailed AuNPs with five sizes range of 14–100 nm were coated by transferrin^28^for three types of cellular exocytosis. The exocytosis experiments were based on 10 h intracellular uptake at 37 °C. The fourth group entailed a zwitterionic D-penicillamine-coated quantum dot (QD)^45^ with size of 8 nm (TEM size), and exocytosis experiments were executed at 37 °C.

To ensure the accuracy of our approach, we do not mix experimental exocytosis data for NPs characterized by different techniques (i.e., TEM and HD) into the same model. For most data, molecular descriptors of the coatings viz., single molecular weight per molecular volume and topological polar surface area per volume, were used to calculate the surface free energy components (the Lifshitz-van de Waals (*γ*^LW^), acid–base (*γ*^AB^), electron acceptor (*γ*^+^) and donor (*γ*^*-*^) terms)^66^ of various NPs according to our previous methods^66^. Values for *γ* of PEG were obtained from measurements by Van Oss et al.^71^. The electrostatic energies (Eq. 7) were obtained from surface potential, which in turn were obtained from zeta potential measured for six charged NPs (see SI Methods). All coating compounds’ identifiers (i.e., SMILE strings) and NPs properties are shown in Tables S3 and S4.

Those data involved six types of cells, namely RAW 264.7 (mouse macrophage), C166 (mouses endothelial), Hela (ovarian cancer cell), U937 (human macrophage), STO (fibroblast) and SNB19 (brain tumor) cells. For the descriptors of cell (membranes) and vesicles, we implemented a surface potential ${\psi }_{0,cell}$ of − 40 mV^66^. The *γ*^LW^_cell_ and *γ*^AB^_cell_ were defined as 39 mJ/m^2^ and 27 mJ/m^2^ for a generic cell surface, respectively. The *γ*^+^_cell_ (2.6 mJ/m^2^) and *γ*^*−*^_cell_ (71 mJ/m^2^) values are calculated based on the previous method^66^.

### Determination of experimental exocytosis rate constant k_exo_

All data described exocytosis involved experiments initiated without NPs in the extracellular medium. Intracellular concentrations NP_t_/NP_0_ of only 16 NPs (12 for U937 cells, four for Hela cells) were recorded over time t. The 16 experiments for U937 and Hela cells were fitted with exocytosis curves $\left( {\left[ {NP} \right]_{{\text{t}}} = \left[ {NP} \right]_{0} \times e^{ - kt} { + }\left[ {NP} \right]_{eq} } \right)$, presented in Fig. S2. A first order kinetic equation $\left( {\left[ {NP} \right]_{{\text{t}}} = \left[ {NP} \right]_{0} \times e^{ - kt} } \right)$ was also used to calculate exocytosis rate constants *k*_*exo*_ of 48 NPs, wherein we took [*NP*]_*eq*_ = 0. The experimental durations of these first and third dataset are 24 and 8 h, respectively. All exocytosis rate constants are given in Table S1.

### Validation of models

We built all models based on neutral NPs in order to reduce the error in surface property changes due to charged NPs reacting with lysosome acid environment, but there are some charged data of U937 to test the error. Six neutral NPs of U937 cells were used to build robust model and another six charged NPs data based on exocytosis experiments, i.e. *k*_*exo*_ values, were used as test data. To test the robustness of the model based on four neutral NPs with exocytosis curves for Hela cells, 14 neutral *k*_*exo*_ (based solely on initial and final NPs concentrations) for Hela cells were used as test data. There is not enough data to test models for RAW 264.7, C166, STO and SNB19 cells.

## Supplementary Information

Supplementary Information.

## Data Availability

All data, models, or code generated or used during the study are available from the corresponding author by request.
