# Supplementary material for: Generic prediction of exocytosis rate constants by size-based surface energies of nanoparticles and cells
Source: Sci Rep. 2022 Oct 24;12:17813. doi: 10.1038/s41598-022-20761-z (PMC9592603; doi:10.1038/s41598-022-20761-z)
Supplement: Supplementary file 1 — Supplementary Information. [file 41598_2022_20761_MOESM1_ESM.docx]

**Generic prediction of exocytosis rate constants by size-based surface energies of nanoparticles and cells**

Bingqing Lu^1*^, Jiaqi Wang ^1^, Paul T. J. Scheepers^2^, A. Jan. Hendriks^1^, Tom M. Nolte^1^

^1^Department of Environmental Science, Institute for Biological and Environmental Sciences, Radboud University Nijmegen, 6500 GL Nijmegen, The Netherlands

^2^Department of Toxicology, Radboud University Nijmegen, 6500 GL Nijmegen, The Netherlands

* Corresponding author: bingqing.lu@ru.nl

**Contents**

1. Methods
2. Equations

Fig S1. The total interaction energy (J) based on three forces over the distance between NPs and vesicles (a) and between vesicles and cells (b)

Fig S2. The fitted time-exocytosis rate curves of 12 gold NPs for U937 cells (a) and four NPs for Hela cells (b).

Table S1. Calculated exocytosis rate constant *k_exocytosis_* for six cell lines and 64 gold NPs.

Table S2. Numbers of NPs per cell surface area based on average size of cells from website.

Table S3. Structure information of NPs coating compounds.

Table S4. Descriptors of NPs for calculating interaction energy between NPs and vesicles.

# 1. Methods

Nolte [^1^](#_ENREF_1) et al. proposed a semi-empirical model to calculate the passive cellular uptake rate of NPs. The model uses properties of NPs and the coating compounds to construct energy diagrams based on three main type of interactions between NPs and cells: Van de Waals forces, electrostatic forces and Lewis acid-base (polar) forces. Their equations are as follows:

|  | (S1) |
| --- | --- |

Where $\Delta G^{LW}$ is the Lifshitz-van de Waals free energy of interaction between NPs in water at distance $h_{0}$, which is the minimum equilibrium distance due to Born repulsion, 0.157 nm (see SI2 for the physicochemical characterization of NPs). $r$ is the particle radius. $h$ is the separation distance between the interacting surfaces and is the characteristic wavelength of the interaction, which is often assumed to be 100 nm.

| $V_{E}\left( h \right)=\frac{\varepsilon\varepsilon_{0}\left( k_{B}T \right)^{2}r}{4e^{2}}{(y^{d})}^{2}\left[ \ln\left( \frac{1+\exp\left( -\kappa h \right)}{1-\exp\left( -\kappa h \right)} \right)+ln(1-\exp\left( -2\kappa h \right)) \right]$ | (S2) |
| --- | --- |

Where $y^{d}$ represents the reduced potential at the Stern layer: , is the potential (V) in the diffuse layer, as approximated via , where $z$ = 0.5 nm [4] and the inverse Debye length $\kappa^{-1}=\sqrt{\frac{\varepsilon\varepsilon_{0}k_{B}T}{2N_{A}Ie^{2}}}$. $k_{B}$ is the Boltzmann constant; 1.38×10^-23^ J/K; $T$ is absolute temperature, 310 K; $e$ is unit charge, 1.602×10^-19^ C; $\varepsilon_{0}$ is the vacuum permittivity, 8.854×10^-12^ CV^-1^m^-1^; $\varepsilon$ is the relative permittivity of water, 78.5; $N_{A}$ is Avogadro’s number, 6.02×10^23^ mol^-1^; $I$ is the ionic strength (M); represents the surface potential of NPs with compounds coating.

|  | (S3) |
| --- | --- |

Where is the correlation length or decay length of the molecules of the liquid medium (for pure water, this value is estimated to be 1 nm [^2^](#_ENREF_2)); $\Delta G^{AB}$ is the polar or acid-base free energy of interaction between NPs at the distance $h_{0}$.

Lifshitz-van de Waals free energies $\Delta G^{LW}$ were determined via the relationship $\Delta G^{LW}=2\left( \sqrt{\gamma_{l}^{LW}}-\sqrt{\gamma^{LW}} \right)\left( \sqrt{\gamma^{LW}}-\sqrt{\gamma_{l}^{LW}} \right)$. Here $\gamma^{LW}$ and $\gamma_{l}^{LW}$ are the Lifshitz-van de Waals surface tensions of the NPs and water, the latter being 21.8 mJ/m^2^ [^3^](#_ENREF_3); Surface potentials of the bare NPs were estimated using the Nernst equation: , where *pH_p.z.c._* is the pH of the medium at which the bare NPs have no net charge; The Lewis acid-base energies $\Delta G^{AB}$ were determined via $\Delta G^{AB}=-4\left[ \sqrt{\left( \gamma_{l}^{+}\gamma_{l}^{-} \right)}+\sqrt{\left( \gamma^{+}\gamma^{-} \right)}-\sqrt{\left( \gamma^{+}\gamma_{l}^{-} \right)}-\sqrt{\left( \gamma^{-}\gamma_{l}^{+} \right)} \right]$ for interaction in water, where $\gamma_{l}^{+}$ and $\gamma_{l}^{-}$ are the electron acceptor and donor terms of water, and $\gamma^{+}$ and $\gamma^{-}$ the electron acceptor and donor terms of the NPs.

1. **Equations**

|  | (S4) |
| --- | --- |

where *t_λ/2_* is the t-value with n-2 degrees of freedom(*λ*=0.05), *n* is the number of samples, Δ*G_0_* is the variables based on the regression of ln(k) and Δ*G*, Δ*G_i_* is the ith samples, $\overline{\Delta G}$is the mean values of all samples, and *S_e_* is the standard error. The 95% confidence intervals of estimated slopes and intercepts were also calculated via:

| $\beta\pm t_{\lambda/2}(n-2)\sqrt{\frac{SS_{\text{e}}/n}{\sum_{i=1}^{n} (\Delta G_{i}-\overline{\Delta G})^{2}}}$ | (S5) |
| --- | --- |
|  | (S6) |

where *SS_e_* is sum of squares due to error.

Table **S1**. Calculated exocytosis rate constant *k_exo_* for six cell lines and 64 gold NPs.

| No. | NPs coating | Cell type | Time,h h | Exocytosis rate, % | *k_exo_* (*10^-6^), /s |
| --- | --- | --- | --- | --- | --- |
| 1 | C1(methoxy) | RAW264.7 | 24 | 18 | 2.2(±0.7) |
| 2 | C6S(hexyl) | RAW264.7 | 24 | 37 | 5.3(±1.0) |
| 3 | C6B-2(3,3-dimethylbutyl) | RAW264.7 | 24 | 24 | 3.2(±0.9) |
| 4 | C6C(cyclohexyl) | RAW264.7 | 24 | 31 | 4.2(±1.4) |
| 5 | C6E-1(5-hexenyl) | RAW264.7 | 24 | 34 | 4.7(±1.4) |
| 6 | C6E-2(2-hexenyl) | RAW264.7 | 24 | 34 | 4.7(±1.6) |
| 7 | C6A(phenyl) | RAW264.7 | 24 | 28 | 3.8(±1.4) |
| 8 | C12S(dodecyl) | RAW264.7 | 24 | 17 | 2.1(±0.5) |
| 9 | C12B(2-butyloctyl) | RAW264.7 | 24 | 10 | 1.2(±0.2) |
| 10 | C12C(cyclododecyl) | RAW264.7 | 24 | 43 | 6.5(±1.4) |
| 11 | C12A-1(5-naphthybutyl) | RAW264.7 | 24 | 18 | 2.2(±0.4) |
| 12 | C12A-2(7-phenylheptyl) | RAW264.7 | 24 | 26 | 3.4(±0.7) |
| 13 | C1(methoxy) | C166 | 24 | 43 | 6.4(±1.0) |
| 14 | C6S(hexyl) | C166 | 24 | 66 | 12.6(±2.4) |
| 15 | C6B-2(3,3-dimethylbutyl) | C166 | 24 | 61 | 11.0(±4.0) |
| 16 | C6C(cyclohexyl) | C166 | 24 | 51 | 8.1(±0.8) |
| 17 | C6E-1(5-hexenyl) | C166 | 24 | 60 | 10.6(±2.5) |
| 18 | C6E-2(2-hexenyl) | C166 | 24 | 49 | 7.8(±4.2) |
| 19 | C6A(phenyl) | C166 | 24 | 51 | 8.1(±1.2) |
| 20 | C12S(dodecyl) | C166 | 24 | 71 | 14.2(±2.4) |
| 21 | C12B(2-butyloctyl) | C166 | 24 | 71 | 14.5(±0.8) |
| 22 | C12C(cyclododecyl) | C166 | 24 | 77 | 17.1(±2.8) |
| 23 | C12A-1(5-naphthylbutyl) | C166 | 24 | 56 | 9.4(±3.4) |
| 24 | C12A-2(7-phenylheptyl) | C166 | 24 | 72 | 14.8(±0.5) |
| 25 | C1(methoxy) | Hela | 24 | 32 | 4.4(±1.6) |
| 26 | C6S(hexyl) | Hela | 24 | 25 | 3.3(±1.2) |
| 27 | C6B-2(3,3-dimethylbutyl) | Hela | 24 | 60 | 10.6(±4.2) |
| 28 | C6C(cyclohexyl) | Hela | 24 | 55 | 9.3(±4.7) |
| 29 | C6E-1(5-hexenyl) | Hela | 24 | 10 | 1.2(±0.6) |
| 30 | C6E-2(2-hexenyl) | Hela | 24 | 65 | 12.2(±2.1) |
| 31 | C6A(phenyl) | Hela | 24 | 57 | 9.8(±3.6) |
| 32 | C12S(dodecyl) | Hela | 24 | 44 | 6.7(±2.5) |
| 33 | C12B(2-butyloctyl) | Hela | 24 | 32 | 4.4(±1.3) |
| 34 | C12C(cyclododecyl) | Hela | 24 | 23 | 3.0(±1.0) |
| 35 | C12A-1(5-naphthylbutyl) | Hela | 24 | 2.1 | 0.2(±0.2) |
| 36 | C12A-2(7-phenylheptyl) | Hela | 24 | 5.6 | 0.7(±0.6) |
| 37 | Citrate | U937 | 48 | 57 | 4.8 |
| 38 | Citrate | U937 | 48 | 46 | 3.6 |
| 39 | Citrate | U937 | 48 | 65 | 6.1 |
| 40 | Cysteamine | U937 | 48 | 23 | 1.5 |
| 41 | Cysteamine | U937 | 48 | 19 | 1.2 |
| 42 | Cysteamine | U937 | 48 | 35 | 2.5 |
| 43 | L-cysteine | U937 | 48 | 67 | 5.0 |
| 44 | L-cysteine | U937 | 48 | 38 | 3.1 |
| 45 | L-cysteine | U937 | 48 | 51 | 4.1 |
| 46 | PEG | U937 | 48 | 83 | 7.4 |
| 47 | PEG | U937 | 48 | 70 | 6.4 |
| 48 | PEG | U937 | 48 | 81 | 1.0 |
| 49 | Transferrin | STO | 8 | 71 | 42.9(±4.9) |
| 50 | Transferrin | STO | 8 | 59 | 31.3(±5.9) |
| 51 | Transferrin | STO | 8 | 50 | 24.3(±1.5) |
| 52 | Transferrin | STO | 8 | 44 | 20.0(±2.1) |
| 53 | Transferrin | STO | 8 | 33 | 13.7(±1.8) |
| 54 | Transferrin | SNB 19 | 8 | 39 | 17.0(±2.2) |
| 55 | Transferrin | SNB 19 | 8 | 24 | 9.6(±2.6) |
| 56 | Transferrin | SNB 19 | 8 | 10 | 3.8(±1.3) |
| 57 | Transferrin | SNB 19 | 8 | 6.3 | 2.2(±0.8) |
| 58 | Transferrin | SNB 19 | 8 | 3.9 | 1.3(±1.7) |
| 59 | Transferrin | Hela | 8 | 38 | 660 |
| 60 | Transferrin | Hela | 8 | 30 | 12.3(±1.5) |
| 61 | Transferrin | Hela | 8 | 18 | 200 |
| 62 | Transferrin | Hela | 8 | 12 | 160 |
| 63 | Transferrin | Hela | 8 | 8.4 | 3.0(±1.9) |
| 64 | D-penicillamine | Hela | 3 | 45 | 480 |

Table S2. Numbers of NPs per cell surface area based on average size of cells.

| NPs | Cells | Initial number of NPs | Average size of cells^a^, μm | Number/cell surface area, μm^-2^ |
| --- | --- | --- | --- | --- |
| C1(methoxy) | RAW264.7 | 88.88888889 | 13.7 | 0.15 |
| C6S(hexyl) | RAW264.7 | 288.8888889 | 13.7 | 0.49 |
| C6B-2(3,3-dimethylbutyl) | RAW264.7 | 177.7777778 | 13.7 | 0.30 |
| C6C(cyclohexyl) | RAW264.7 | 133.3333333 | 13.7 | 0.23 |
| C6E-1(5-hexenyl) | RAW264.7 | 133.3333333 | 13.7 | 0.23 |
| C6E-2(2-hexenyl) | RAW264.7 | 200 | 13.7 | 0.34 |
| C6A(phenyl) | RAW264.7 | 244.4444444 | 13.7 | 0.41 |
| C12S(dodecyl) | RAW264.7 | 244.4444444 | 13.7 | 0.41 |
| C12B(2-butyloctyl) | RAW264.7 | 2300 | 13.7 | 3.90 |
| C12C(cyclododecyl) | RAW264.7 | 133.3333333 | 13.7 | 0.23 |
| C12A-1(5-naphthybutyl) | RAW264.7 | 155.5555556 | 13.7 | 0.26 |
| C12A-2(7-phenylheptyl) | RAW264.7 | 111.1111111 | 13.7 | 0.19 |
| C1(methoxy) | C166 | 111.1111111 | Length 40, width 20, thickness 5 | 0.19 |
| C6S(hexyl) | C166 | 58.82352941 | Length 40, width 20, thickness 5 | 0.03 |
| C6B-2(3,3-dimethylbutyl) | C166 | 627.4509804 | Length 40, width 20, thickness 5 | 0.29 |
| C6C(cyclohexyl) | C166 | 98.03921569 | Length 40, width 20, thickness 5 | 0.05 |
| C6E-1(5-hexenyl) | C166 | 156.8627451 | Length 40, width 20, thickness 5 | 0.07 |
| C6E-2(2-hexenyl) | C166 | 117.6470588 | Length 40, width 20, thickness 5 | 0.05 |
| C6A(phenyl) | C166 | 117.6470588 | Length 40, width 20, thickness 5 | 0.05 |
| C12S(dodecyl) | C166 | 176.4705882 | Length 40, width 20, thickness 5 | 0.08 |
| C12B(2-butyloctyl) | C166 | 176.4705882 | Length 40, width 20, thickness 5 | 0.08 |
| C12C(cyclododecyl) | C166 | 23000 | Length 40, width 20, thickness 5 | 10.70 |
| C12A-1(5-naphthybutyl) | C166 | 470.5882353 | Length 40, width 20, thickness 5 | 0.22 |
| C12A-2(7-phenylheptyl) | C166 | 823.5294118 | Length 40, width 20, thickness 5 | 0.38 |
| C1(methoxy) | Hela | 411.7647059 | 30 | 0.19 |
| C6S(hexyl) | Hela | 411.7647059 | 30 | 0.19 |
| C6B-2(3,3-dimethylbutyl) | Hela | 409.0909091 | 30 | 0.14 |
| C6C(cyclohexyl) | Hela | 1363.636364 | 30 | 0.48 |
| C6E-1(5-hexenyl) | Hela | 272.7272727 | 30 | 0.10 |
| C6E-2(2-hexenyl) | Hela | 500 | 30 | 0.18 |
| C6A(phenyl) | Hela | 363.6363636 | 30 | 0.13 |
| C12S(dodecyl) | Hela | 1636.363636 | 30 | 0.58 |
| C12B(2-butyloctyl) | Hela | 363.6363636 | 30 | 0.13 |
| C12C(cyclododecyl) | Hela | 500 | 30 | 0.18 |
| C12A-1(5-naphthybutyl) | Hela | 13000 | 30 | 4.60 |
| C12A-2(7-phenylheptyl) | Hela | 70 | 30 | 0.02 |
| Transferrin | Hela | 400 | 30 | 0.19 |
| Transferrin | Hela | 1408.695652 | 30 | 0.66 |
| Transferrin | Hela | 2452.173913 | 30 | 1.14 |
| Transferrin | Hela | 1930.434783 | 30 | 0.90 |
| Transferrin | Hela | 1182.608696 | 30 | 0.55 |
| D-penicillamine | Hela | b | 30 | b |
| L-cysteine | U937 | 711111.1111 | 21 | 513.44 |
| L-cysteine | U937 | 1111111.111 | 21 | 802.25 |
| L-cysteine | U937 | 24324.32432 | 21 | 17.56 |
| MPEG43-sulfhydryl | U937 | 266666.6667 | 21 | 192.54 |
| MPEG43-sulfhydryl | U937 | 844444.4444 | 21 | 609.71 |
| MPEG43-sulfhydryl | U937 | 18918.91892 | 21 | 13.66 |
| Transferrin | STO | 191.3043478 | 1 | 60.92 |
| Transferrin | STO | 260.8695652 | 1 | 83.08 |
| Transferrin | STO | 469.5652174 | 1 | 149.54 |
| Transferrin | STO | 347.826087 | 1 | 110.77 |
| Transferrin | STO | 156.5217391 | 1 | 49.85 |
| Transferrin | SNB 19 | 417.3913043 | 13.6 | 0.72 |
| Transferrin | SNB 19 | 782.6086957 | 13.6 | 1.35 |
| Transferrin | SNB 19 | 1286.956522 | 13.6 | 2.22 |
| Transferrin | SNB 19 | 869.5652174 | 13.6 | 1.50 |
| Transferrin | SNB 19 | 365.2173913 | 13.6 | 0.63 |

^a^ denotes average size of cells come from google to give a rough reference.

^b^ denotes no data has been found.

Table S3. Structure information of NPs coating compounds.

| No. | Compounds | Size nm | SMILE | Ref |
| --- | --- | --- | --- | --- |
| 1 | C1(methoxy) | 25.30 | OCCOCC(=O)NC | [^4^](#_ENREF_4) |
| 2 | C6S(hexyl) | 25.60 | OCCOCC(=O)NCCCCCC | [^4^](#_ENREF_4) |
| 3 | C6B-2(3,3-dimethylbutyl) | 25.90 | OCCOCC(=O)NCCC(C)(C)C | [^4^](#_ENREF_4) |
| 4 | C6C(cyclohexyl) | 25.17 | OCCOCC(=O)NC1CCCCC1 | [^4^](#_ENREF_4) |
| 5 | C6E-1(5-hexenyl) | 25.17 | OCCOCC(=O)NCCCCC=C | [^4^](#_ENREF_4) |
| 6 | C6E-2(2-hexenyl) | 26.73 | OCCOCC(=O)NCC=CCCC | [^4^](#_ENREF_4) |
| 7 | C6A(phenyl) | 28.13 | OCCOCC(=O)NC1=CC=CC=C1 | [^4^](#_ENREF_4) |
| 8 | C12S(dodecyl) | 27.53 | OCCOCC(=O)NCCCCCCCCCCCC | [^4^](#_ENREF_4) |
| 9 | C12B(2-butyloctyl) | 27.43 | OCCOCC(=O)NCC(CCCC)CCCCCC | [^4^](#_ENREF_4) |
| 10 | C12C(cyclododecyl) | 25.80 | OCCOCC(=O)NC1CCCCCCCCCCC1 | [^4^](#_ENREF_4) |
| 11 | C12A-1(5-naphthybutyl) | 27.00 | OCCOCC(=O)NCCC1=CC2=CC=CC=C2C=C1 | [^4^](#_ENREF_4) |
| 12 | C12A-2(7-phenylheptyl) | 25.90 | OCCOCC(=O)NCCCCCCc1ccccc1 | [^4^](#_ENREF_4) |
| 13 | C1(methoxy) | 25.30 | OCCOCC(=O)NC | [^4^](#_ENREF_4) |
| 14 | C6S(hexyl) | 25.60 | OCCOCC(=O)NCCCCCC | [^4^](#_ENREF_4) |
| 15 | C6B-2(3,3-dimethylbutyl) | 25.90 | OCCOCC(=O)NCCC(C)(C)C | [^4^](#_ENREF_4) |
| 16 | C6C(cyclohexyl) | 25.17 | OCCOCC(=O)NC1CCCCC1 | [^4^](#_ENREF_4) |
| 17 | C6E-1(5-hexenyl) | 25.17 | OCCOCC(=O)NCCCCC=C | [^4^](#_ENREF_4) |
| 18 | C6E-2(2-hexenyl) | 26.73 | OCCOCC(=O)NCC=CCCC | [^4^](#_ENREF_4) |
| 19 | C6A(phenyl) | 28.13 | OCCOCC(=O)NC1=CC=CC=C1 | [^4^](#_ENREF_4) |
| 20 | C12S(dodecyl) | 27.53 | OCCOCC(=O)NCCCCCCCCCCCC | [^4^](#_ENREF_4) |
| 21 | C12B(2-butyloctyl) | 27.43 | OCCOCC(=O)NCC(CCCC)CCCCCC | [^4^](#_ENREF_4) |
| 22 | C12C(cyclododecyl) | 25.80 | OCCOCC(=O)NC1CCCCCCCCCCC1 | [^4^](#_ENREF_4) |
| 23 | C12A-1(5-naphthybutyl) | 27.00 | OCCOCC(=O)NCCC1=CC2=CC=CC=C2C=C1 | [^4^](#_ENREF_4) |
| 24 | C12A-2(7-phenylheptyl) | 25.90 | OCCOCC(=O)NCCCCCCc1ccccc1 | [^4^](#_ENREF_4) |
| 25 | C1(methoxy) | 25.30 | OCCOCC(=O)NC | [^4^](#_ENREF_4) |
| 26 | C6S(hexyl) | 25.60 | OCCOCC(=O)NCCCCCC | [^4^](#_ENREF_4) |
| 27 | C6B-2(3,3-dimethylbutyl) | 25.90 | OCCOCC(=O)NCCC(C)(C)C | [^4^](#_ENREF_4) |
| 28 | C6C(cyclohexyl) | 25.17 | OCCOCC(=O)NC1CCCCC1 | [^4^](#_ENREF_4) |
| 29 | C6E-1(5-hexenyl) | 25.17 | OCCOCC(=O)NCCCCC=C | [^4^](#_ENREF_4) |
| 30 | C6E-2(2-hexenyl) | 26.73 | OCCOCC(=O)NCC=CCCC | [^4^](#_ENREF_4) |
| 31 | C6A(phenyl) | 28.13 | OCCOCC(=O)NC1=CC=CC=C1 | [^4^](#_ENREF_4) |
| 32 | C12S(dodecyl) | 27.53 | OCCOCC(=O)NCCCCCCCCCCCC | [^4^](#_ENREF_4) |
| 33 | C12B(2-butyloctyl) | 27.43 | OCCOCC(=O)NCC(CCCC)CCCCCC | [^4^](#_ENREF_4) |
| 34 | C12C(cyclododecyl) | 25.80 | OCCOCC(=O)NC1CCCCCCCCCCC1 | [^4^](#_ENREF_4) |
| 35 | C12A-1(5-naphthybutyl) | 27.00 | OCCOCC(=O)NCCC1=CC2=CC=CC=C2C=C1 | [^4^](#_ENREF_4) |
| 36 | C12A-2(7-phenylheptyl) | 25.90 | OCCOCC(=O)NCCCCCCc1ccccc1 | [^4^](#_ENREF_4) |
| 37 | Citrate | 14.41 | C(C(=O)[O-])C(CC(=O)[O-])(C(=O)[O-])O | [^5^](#_ENREF_5) |
| 38 | Citrate | 26.86 | C(C(=O)[O-])C(CC(=O)[O-])(C(=O)[O-])O | [^5^](#_ENREF_5) |
| 39 | Citrate | 45.49 | C(C(=O)[O-])C(CC(=O)[O-])(C(=O)[O-])O | [^5^](#_ENREF_5) |
| 40 | Cysteamine | 13.48 | C(CS)N | [^5^](#_ENREF_5) |
| 41 | Cysteamine | 29.02 | C(CS)N | [^5^](#_ENREF_5) |
| 42 | Cysteamine | 47.79 | C(CS)N | [^5^](#_ENREF_5) |
| 43 | L-cysteine | 16.26 | C(C(C(=O)O)N)S | [^5^](#_ENREF_5) |
| 44 | L-cysteine | 27.23 | C(C(C(=O)O)N)S | [^5^](#_ENREF_5) |
| 45 | L-cysteine | 48.84 | C(C(C(=O)O)N)S | [^5^](#_ENREF_5) |
| 46 | PEG | 25.42 | [OCCO] | [^5^](#_ENREF_5) |
| 47 | PEG | 35.93 | [OCCO] | [^5^](#_ENREF_5) |
| 48 | PEG | 55.15 | [OCCO] | [^5^](#_ENREF_5) |
| 49 | transferrin | 14.00 | CC(CC(N([H])C(C(N([H])C(C(N([H])C(C(N([H])C(C(N([H])C(C(N([H])C(C(N([H])C(C([N+]([H])([H])[H])C)=O)CC([O-])=O)=O)CCCN([H])C(N([H])[H])=[N+]([H])[H])=O)CC([O-])=O)=O)CCC(N([H])[H])=O)=O)CC1C=CC(O[H])=CC=10)=O)CCC([O-])=O)=O)C(N([H])C(C(N([H])C(C(N([H])C(C(N([H])C(C(N([H])C(C(N([H])C(C(N([H])C(C([O-])=O)CCCN([H])/C(/N([H])[H])=[N+](\[H])/[H])=O)C(O[H])C)=O)CC(N([H])[H])=O)=O)CC([O-])=O)=O)CC(C)C)=O)CS[H])=O)CC(C)C)=O)C | [^6^](#_ENREF_6) |
| 50 | transferrin | 30.00 | CC(CC(N([H])C(C(N([H])C(C(N([H])C(C(N([H])C(C(N([H])C(C(N([H])C(C(N([H])C(C([N+]([H])([H])[H])C)=O)CC([O-])=O)=O)CCCN([H])C(N([H])[H])=[N+]([H])[H])=O)CC([O-])=O)=O)CCC(N([H])[H])=O)=O)CC1C=CC(O[H])=CC=1)=O)CCC([O-])=O)=O)C(N([H])C(C(N([H])C(C(N([H])C(C(N([H])C(C(N([H])C(C(N([H])C(C(N([H])C(C([O-])=O)CCCN([H])/C(/N([H])[H])=[N+](\[H])/[H])=O)C(O[H])C)=O)CC(N([H])[H])=O)=O)CC([O-])=O)=O)CC(C)C)=O)CS[H])=O)CC(C)C)=O)C | [^6^](#_ENREF_6) |
| 51 | transferrin | 50.00 | CC(CC(N([H])C(C(N([H])C(C(N([H])C(C(N([H])C(C(N([H])C(C(N([H])C(C(N([H])C(C([N+]([H])([H])[H])C)=O)CC([O-])=O)=O)CCCN([H])C(N([H])[H])=[N+]([H])[H])=O)CC([O-])=O)=O)CCC(N([H])[H])=O)=O)CC1C=CC(O[H])=CC=1)=O)CCC([O-])=O)=O)C(N([H])C(C(N([H])C(C(N([H])C(C(N([H])C(C(N([H])C(C(N([H])C(C(N([H])C(C([O-])=O)CCCN([H])/C(/N([H])[H])=[N+](\[H])/[H])=O)C(O[H])C)=O)CC(N([H])[H])=O)=O)CC([O-])=O)=O)CC(C)C)=O)CS[H])=O)CC(C)C)=O)C | [^6^](#_ENREF_6) |
| 52 | transferrin | 74.00 | CC(CC(N([H])C(C(N([H])C(C(N([H])C(C(N([H])C(C(N([H])C(C(N([H])C(C(N([H])C(C([N+]([H])([H])[H])C)=O)CC([O-])=O)=O)CCCN([H])C(N([H])[H])=[N+]([H])[H])=O)CC([O-])=O)=O)CCC(N([H])[H])=O)=O)CC1C=CC(O[H])=CC=1)=O)CCC([O-])=O)=O)C(N([H])C(C(N([H])C(C(N([H])C(C(N([H])C(C(N([H])C(C(N([H])C(C(N([H])C(C([O-])=O)CCCN([H])/C(/N([H])[H])=[N+](\[H])/[H])=O)C(O[H])C)=O)CC(N([H])[H])=O)=O)CC([O-])=O)=O)CC(C)C)=O)CS[H])=O)CC(C)C)=O)C | [^6^](#_ENREF_6) |
| 53 | transferrin | 100.00 | CC(CC(N([H])C(C(N([H])C(C(N([H])C(C(N([H])C(C(N([H])C(C(N([H])C(C(N([H])C(C([N+]([H])([H])[H])C)=O)CC([O-])=O)=O)CCCN([H])C(N([H])[H])=[N+]([H])[H])=O)CC([O-])=O)=O)CCC(N([H])[H])=O)=O)CC1C=CC(O[H])=CC=1)=O)CCC([O-])=O)=O)C(N([H])C(C(N([H])C(C(N([H])C(C(N([H])C(C(N([H])C(C(N([H])C(C(N([H])C(C([O-])=O)CCCN([H])/C(/N([H])[H])=[N+](\[H])/[H])=O)C(O[H])C)=O)CC(N([H])[H])=O)=O)CC([O-])=O)=O)CC(C)C)=O)CS[H])=O)CC(C)C)=O)C | [^6^](#_ENREF_6) |
| 54 | transferrin | 14.00 | CC(CC(N([H])C(C(N([H])C(C(N([H])C(C(N([H])C(C(N([H])C(C(N([H])C(C(N([H])C(C([N+]([H])([H])[H])C)=O)CC([O-])=O)=O)CCCN([H])C(N([H])[H])=[N+]([H])[H])=O)CC([O-])=O)=O)CCC(N([H])[H])=O)=O)CC1C=CC(O[H])=CC=1)=O)CCC([O-])=O)=O)C(N([H])C(C(N([H])C(C(N([H])C(C(N([H])C(C(N([H])C(C(N([H])C(C(N([H])C(C([O-])=O)CCCN([H])/C(/N([H])[H])=[N+](\[H])/[H])=O)C(O[H])C)=O)CC(N([H])[H])=O)=O)CC([O-])=O)=O)CC(C)C)=O)CS[H])=O)CC(C)C)=O)C | [^6^](#_ENREF_6) |
| 55 | transferrin | 30.00 | CC(CC(N([H])C(C(N([H])C(C(N([H])C(C(N([H])C(C(N([H])C(C(N([H])C(C(N([H])C(C([N+]([H])([H])[H])C)=O)CC([O-])=O)=O)CCCN([H])C(N([H])[H])=[N+]([H])[H])=O)CC([O-])=O)=O)CCC(N([H])[H])=O)=O)CC1C=CC(O[H])=CC=1)=O)CCC([O-])=O)=O)C(N([H])C(C(N([H])C(C(N([H])C(C(N([H])C(C(N([H])C(C(N([H])C(C(N([H])C(C([O-])=O)CCCN([H])/C(/N([H])[H])=[N+](\[H])/[H])=O)C(O[H])C)=O)CC(N([H])[H])=O)=O)CC([O-])=O)=O)CC(C)C)=O)CS[H])=O)CC(C)C)=O)C | [^6^](#_ENREF_6) |
| 56 | transferrin | 50.00 | CC(CC(N([H])C(C(N([H])C(C(N([H])C(C(N([H])C(C(N([H])C(C(N([H])C(C(N([H])C(C([N+]([H])([H])[H])C)=O)CC([O-])=O)=O)CCCN([H])C(N([H])[H])=[N+]([H])[H])=O)CC([O-])=O)=O)CCC(N([H])[H])=O)=O)CC1C=CC(O[H])=CC=1)=O)CCC([O-])=O)=O)C(N([H])C(C(N([H])C(C(N([H])C(C(N([H])C(C(N([H])C(C(N([H])C(C(N([H])C(C([O-])=O)CCCN([H])/C(/N([H])[H])=[N+](\[H])/[H])=O)C(O[H])C)=O)CC(N([H])[H])=O)=O)CC([O-])=O)=O)CC(C)C)=O)CS[H])=O)CC(C)C)=O)C | [^6^](#_ENREF_6) |
| 57 | transferrin | 74.00 | CC(CC(N([H])C(C(N([H])C(C(N([H])C(C(N([H])C(C(N([H])C(C(N([H])C(C(N([H])C(C([N+]([H])([H])[H])C)=O)CC([O-])=O)=O)CCCN([H])C(N([H])[H])=[N+]([H])[H])=O)CC([O-])=O)=O)CCC(N([H])[H])=O)=O)CC1C=CC(O[H])=CC=2)=O)CCC([O-])=O)=O)C(N([H])C(C(N([H])C(C(N([H])C(C(N([H])C(C(N([H])C(C(N([H])C(C(N([H])C(C([O-])=O)CCCN([H])/C(/N([H])[H])=[N+](\[H])/[H])=O)C(O[H])C)=O)CC(N([H])[H])=O)=O)CC([O-])=O)=O)CC(C)C)=O)CS[H])=O)CC(C)C)=O)C | [^6^](#_ENREF_6) |
| 58 | transferrin | 100.00 | CC(CC(N([H])C(C(N([H])C(C(N([H])C(C(N([H])C(C(N([H])C(C(N([H])C(C(N([H])C(C([N+]([H])([H])[H])C)=O)CC([O-])=O)=O)CCCN([H])C(N([H])[H])=[N+]([H])[H])=O)CC([O-])=O)=O)CCC(N([H])[H])=O)=O)CC1C=CC(O[H])=CC=3)=O)CCC([O-])=O)=O)C(N([H])C(C(N([H])C(C(N([H])C(C(N([H])C(C(N([H])C(C(N([H])C(C(N([H])C(C([O-])=O)CCCN([H])/C(/N([H])[H])=[N+](\[H])/[H])=O)C(O[H])C)=O)CC(N([H])[H])=O)=O)CC([O-])=O)=O)CC(C)C)=O)CS[H])=O)CC(C)C)=O)C | [^6^](#_ENREF_6) |
| 59 | transferrin | 14.00 | CC(CC(N([H])C(C(N([H])C(C(N([H])C(C(N([H])C(C(N([H])C(C(N([H])C(C(N([H])C(C([N+]([H])([H])[H])C)=O)CC([O-])=O)=O)CCCN([H])C(N([H])[H])=[N+]([H])[H])=O)CC([O-])=O)=O)CCC(N([H])[H])=O)=O)CC1C=CC(O[H])=CC=4)=O)CCC([O-])=O)=O)C(N([H])C(C(N([H])C(C(N([H])C(C(N([H])C(C(N([H])C(C(N([H])C(C(N([H])C(C([O-])=O)CCCN([H])/C(/N([H])[H])=[N+](\[H])/[H])=O)C(O[H])C)=O)CC(N([H])[H])=O)=O)CC([O-])=O)=O)CC(C)C)=O)CS[H])=O)CC(C)C)=O)C | [^6^](#_ENREF_6) |
| 60 | transferrin | 30.00 | CC(CC(N([H])C(C(N([H])C(C(N([H])C(C(N([H])C(C(N([H])C(C(N([H])C(C(N([H])C(C([N+]([H])([H])[H])C)=O)CC([O-])=O)=O)CCCN([H])C(N([H])[H])=[N+]([H])[H])=O)CC([O-])=O)=O)CCC(N([H])[H])=O)=O)CC1C=CC(O[H])=CC=5)=O)CCC([O-])=O)=O)C(N([H])C(C(N([H])C(C(N([H])C(C(N([H])C(C(N([H])C(C(N([H])C(C(N([H])C(C([O-])=O)CCCN([H])/C(/N([H])[H])=[N+](\[H])/[H])=O)C(O[H])C)=O)CC(N([H])[H])=O)=O)CC([O-])=O)=O)CC(C)C)=O)CS[H])=O)CC(C)C)=O)C | [^6^](#_ENREF_6) |
| 61 | transferrin | 50.00 | CC(CC(N([H])C(C(N([H])C(C(N([H])C(C(N([H])C(C(N([H])C(C(N([H])C(C(N([H])C(C([N+]([H])([H])[H])C)=O)CC([O-])=O)=O)CCCN([H])C(N([H])[H])=[N+]([H])[H])=O)CC([O-])=O)=O)CCC(N([H])[H])=O)=O)CC1C=CC(O[H])=CC=6)=O)CCC([O-])=O)=O)C(N([H])C(C(N([H])C(C(N([H])C(C(N([H])C(C(N([H])C(C(N([H])C(C(N([H])C(C([O-])=O)CCCN([H])/C(/N([H])[H])=[N+](\[H])/[H])=O)C(O[H])C)=O)CC(N([H])[H])=O)=O)CC([O-])=O)=O)CC(C)C)=O)CS[H])=O)CC(C)C)=O)C | [^6^](#_ENREF_6) |
| 62 | transferrin | 74.00 | CC(CC(N([H])C(C(N([H])C(C(N([H])C(C(N([H])C(C(N([H])C(C(N([H])C(C(N([H])C(C([N+]([H])([H])[H])C)=O)CC([O-])=O)=O)CCCN([H])C(N([H])[H])=[N+]([H])[H])=O)CC([O-])=O)=O)CCC(N([H])[H])=O)=O)CC1C=CC(O[H])=CC=7)=O)CCC([O-])=O)=O)C(N([H])C(C(N([H])C(C(N([H])C(C(N([H])C(C(N([H])C(C(N([H])C(C(N([H])C(C([O-])=O)CCCN([H])/C(/N([H])[H])=[N+](\[H])/[H])=O)C(O[H])C)=O)CC(N([H])[H])=O)=O)CC([O-])=O)=O)CC(C)C)=O)CS[H])=O)CC(C)C)=O)C | [^6^](#_ENREF_6) |
| 63 | transferrin | 100.00 | CC(CC(N([H])C(C(N([H])C(C(N([H])C(C(N([H])C(C(N([H])C(C(N([H])C(C(N([H])C(C([N+]([H])([H])[H])C)=O)CC([O-])=O)=O)CCCN([H])C(N([H])[H])=[N+]([H])[H])=O)CC([O-])=O)=O)CCC(N([H])[H])=O)=O)CC1C=CC(O[H])=CC=8)=O)CCC([O-])=O)=O)C(N([H])C(C(N([H])C(C(N([H])C(C(N([H])C(C(N([H])C(C(N([H])C(C(N([H])C(C([O-])=O)CCCN([H])/C(/N([H])[H])=[N+](\[H])/[H])=O)C(O[H])C)=O)CC(N([H])[H])=O)=O)CC([O-])=O)=O)CC(C)C)=O)CS[H])=O)CC(C)C)=O)C | [^6^](#_ENREF_6) |
| 64 | D-penicillamine | 8.00 | CC(C)(C(C(=O)O)N)S | [^7^](#_ENREF_7) |

Table S4. Descriptors of NPs for calculating interaction energy between NPs and vesicles.

| NPs | TPSA | MW | Volume | TPSA/  Volume | MW/  Volume | γ^LW^  mJ/m^2^ | γ^AB^ mJ/m^2^ | γ^+^  mJ/m^2^ | γ^-^  mJ/m^2^ | Surface potential mV |
| --- | --- | --- | --- | --- | --- | --- | --- | --- | --- | --- |
| C1(methoxy) | 58.6 | 133.2 | 128.0 | 0.5 | 1.0 | 32.7 | 12.0 | 1.2 | 31.2 | 0 |
| C6S(hexyl) | 58.6 | 203.3 | 212.0 | 0.3 | 1.0 | 29.3 | 6.3 | 0.6 | 16.3 | 0 |
| C6B-2(3,3-dimethylbutyl) | 58.6 | 203.3 | 211.0 | 0.3 | 1.0 | 29.5 | 6.4 | 0.6 | 16.5 | 0 |
| C6C(cyclohexyl) | 58.6 | 201.3 | 201.4 | 0.3 | 1.0 | 31.0 | 6.8 | 0.7 | 17.5 | 0 |
| C6E-1(5-hexenyl) | 58.6 | 201.3 | 206.4 | 0.3 | 1.0 | 30.0 | 6.6 | 0.6 | 17.0 | 0 |
| C6E-2(2-hexenyl) | 58.6 | 201.3 | 205.8 | 0.3 | 1.0 | 30.1 | 6.6 | 0.6 | 17.0 | 0 |
| C6A(phenyl) | 58.6 | 195.2 | 182.8 | 0.3 | 1.1 | 33.8 | 7.7 | 0.7 | 20.0 | 0 |
| C12S(dodecyl) | 58.6 | 287.4 | 312.8 | 0.2 | 0.9 | 27.7 | 3.5 | 0.3 | 9.0 | 0 |
| C12B(2-butyloctyl) | 58.6 | 287.4 | 312.6 | 0.2 | 0.9 | 27.7 | 3.5 | 0.3 | 9.0 | 0 |
| C12C(cyclododecyl) | 58.6 | 285.4 | 302.2 | 0.2 | 0.9 | 28.7 | 3.7 | 0.4 | 9.6 | 0 |
| C12A-1(5-naphthybutyl) | 58.6 | 273.3 | 260.4 | 0.2 | 1.0 | 33.0 | 4.7 | 0.5 | 12.2 | 0 |
| C12A-2(7-phenylheptyl) | 58.6 | 279.4 | 283.7 | 0.2 | 1.0 | 30.4 | 4.1 | 0.4 | 10.6 | 0 |
| Citrate | 140.6 | 189.1 | 143.5 | 1.0 | 1.3 | 44.0 | 28.4 | 2.7 | 74.1 | -31.2 |
| Citrate | 140.6 | 189.1 | 143.5 | 1.0 | 1.3 | 44.0 | 28.4 | 2.7 | 74.1 | -40.3 |
| Citrate | 140.6 | 189.1 | 143.5 | 1.0 | 1.3 | 44.0 | 28.4 | 2.7 | 74.1 | -36.9 |
| Cysteamine | 26.0 | 78.0 | 75.2 | 0.3 | 1.0 | 32.5 | 8.5 | 0.8 | 22.1 | 28.1 |
| Cysteamine | 26.0 | 78.0 | 75.2 | 0.3 | 1.0 | 32.5 | 8.5 | 0.8 | 22.1 | 49.3 |
| Cysteamine | 26.0 | 78.0 | 75.2 | 0.3 | 1.0 | 32.5 | 8.5 | 0.8 | 22.1 | 41.6 |
| L-cysteine | 63.3 | 121.0 | 102.2 | 0.6 | 1.2 | 38.5 | 17.1 | 1.6 | 44.6 | 0 |
| PEG | 40.46 | 62.07 | 62.27 | 0.7 | 1.0 | 43 | * | 0.43 | 64 [^8^](#_ENREF_8) | 0 |
| transferrin | 889.58 | 1822.98 | 1619.89 | 0.55 | 1.13 | 36.14 | 14.90 | 1.43 | 38.78 | 0 |
| D-penicillamine | 63.3 | 135.0 | 149.2 | 0.5 | 1.1 | 35.3 | 12.4 | 1.2 | 32.2 | 0 |

.

|  |  |
| --- | --- |

Fig S1. The total interaction energy (J) based on three forces over the distance between NPs and vesicles (a) and between vesicles and cells (b).

|  |  |
| --- | --- |

Fig S2. The fitted time-exocytosis rate curves of 12 gold NPs for U937 cells ^5^ (a) and four NPs for Hela cells ^6, 7^ (b).

# Reference

1 Nolte, T. M., Kettler, K., Meesters, J. A. J., Hendriks, A. J. & van de Meent, D. A semi-empirical model for transport of inorganic nanoparticles across a lipid bilayer: implications for uptake by living cells. *Environ. Toxicol. Chem.* **34**, 488-496, doi:10.1002/etc.2812 (2015).

2 Van Oss, C. J. Long-range and short-range mechanisms of hydrophobic attraction and hydrophilic repulsion in specific and aspecific interactions. *J. Mol. Recognit.* **16**, 177-190, doi:10.1002/jmr.618 (2003).

3 Brant, J. A. & Childress, A. E. Assessing short-range membrane-colloid interactions using surface energetics. *J. Membr. Sci.* **203**, 257-273, doi:10.1016/s0376-7388(02)00014-5 (2002).

4 Ho, L. W. C., Yin, B. H., Dai, G. L. & Choi, C. H. J. Effect of Surface Modification with Hydrocarbyl Groups on the Exocytosis of Nanoparticles. *Biochemistry* **60**, 1019-1030, doi:10.1021/acs.biochem.0c00631 (2021).

5 Oh, N. & Park, J. H. Surface Chemistry of Gold Nanoparticles Mediates Their Exocytosis in Macrophages. *Acs Nano* **8**, 6232-6241, doi:10.1021/nn501668a (2014).

6 Chithrani, B. D. & Chan, W. C. W. Elucidating the mechanism of cellular uptake and removal of protein-coated gold nanoparticles of different sizes and shapes. *Nano Lett.* **7**, 1542-1550, doi:10.1021/nl070363y (2007).

7 Jiang, X. E. *et al.* Endo- and Exocytosis of Zwitterionic Quantum Dot Nanoparticles by Live HeLa Cells. *Acs Nano* **4**, 6787-6797, doi:10.1021/nn101277w (2010).

8 Vanoss, C. J., Chaudhury, M. K. & Good, R. J. MONOPOLAR SURFACES. *Adv. Colloid Interface Sci.* **28**, 35-64, doi:10.1016/0001-8686(87)80008-8 (1987).
